# Population density shapes patterns of survival and reproduction in *Eleutheria dichotoma* (Hydrozoa: Anthoathecata)

**DOI:** 10.1007/s00227-018-3309-z

**Published:** 2018-02-17

**Authors:** Aleksandra Dańko, Ralf Schaible, Joanna Pijanowska, Maciej J. Dańko

**Affiliations:** 1 Laboratory of Evolutionary Biodemography, Max Planck Institute for Demographic Research, Konrad-Zuse-Strasse 1, 18057 Rostock, Germany; 2 Department of Hydrobiology, University of Warsaw, Żwirki i Wigury 101, 02-089 Warsaw, Poland

## Abstract

Budding hydromedusae have high reproductive rates due to asexual reproduction and can occur in high population densities along the coasts, specifically in tidal pools. In laboratory experiments, we investigated the effects of population density on the survival and reproductive strategies of a single clone of *Eleutheria dichotoma*. We found that sexual reproduction occurs with the highest rate at medium population densities. Increased sexual reproduction was associated with lower budding (asexual reproduction) and survival probability. Sexual reproduction results in the production of motile larvae that can, in contrast to medusae, seek to escape unfavorable conditions by actively looking for better environments. The successful settlement of a larva results in starting the polyp stage, which is probably more resistant to environmental conditions. This is the first study that has examined the life-history strategies of the budding hydromedusa *E. dichotoma* by conducting a long-term experiment with a relatively large sample size that allowed for the examination of age-specific mortality and reproductive rates. We found that most sexual and asexual reproduction occurred at the beginning of life following a very rapid process of maturation. The parametric models fitted to the mortality data showed that population density was associated with an increase in the rate of aging, an increase in the level of late-life mortality plateau, and a decrease in the hidden heterogeneity in individual mortality rates. The effects of population density on life-history traits are discussed in the context of resource allocation and the r/K-strategies’ continuum concept.

**Electronic supplementary material:**

The online version of this article (10.1007/s00227-018-3309-z) contains supplementary material, which is available to authorized users.

## Introduction

Population density is an important environmental factor that can affect the fitness-related traits of an individual. Increased population density can cause resource depletion, increased stress from intra- and interspecific competition, and increased accumulation of harmful metabolites. Density-related factors can activate both direct and indirect mechanisms of somatic deterioration, which may, in turn, result in declining performance with age. In particular, population density can trigger a change in resource allocations strategies, and may affect life-history traits such as growth rate, age at maturity, adult body size, and lifespan (Peters and Barbosa 1977; Kozłowski 1992; Stearns 1992; Dańko et al. 2015, 2017a).

Density dependence is closely associated with the classic models of life-history evolution: r- and K-selection. In short, r-selection occurs mainly among populations that generally remain sufficiently small to avoid the effects of density dependence. In general, r-selection should occur among populations that are characterized by rapid growth, a high degree of dispersal, high levels of reproductive effort, a tendency to mature early, a small average body size, and closely spaced generations. K-selection, in contrast, should occur among populations that are near carrying capacity; that live in resource-limited, competitive environments; and that are subject to strong density effects. In the modern view, K-selected populations employ a variety of strategies, some of which would fit better in the r-selection than in the K-selection model (Stearns 1992; Reznick et al. 2002; Kozłowski 2006). Nonetheless, it seems clear that populations with initially low levels of regulation via density dependence should reproduce more rapidly, as the rapid propagation of a genotype is the most effective strategy for increasing fitness (which is analogous to the r-strategist concept). On the other hand, when density levels are high, an organism should invest more resources in growth and tissue maintenance in order to maximize quality, and thus in future reproduction in a competitive environment (K-strategists’ concept; see also Pianka 1970; Stearns 1992; Kozłowski 2006; Dańko et al. 2017a).

In natural habitats, resources are typically unequally partitioned among individuals, and this inequality increases when resources become scarce; i.e., due to increased density. Łomnicki (1978) showed that unequal resource partitioning is a source of variation among individuals that ensures successful emigration to new areas. Clearly, intraspecific competition for resources placed additional stress on a population. Intraspecific competition can be partially or fully avoided by complex life-cycles (Łomnicki 1988), in which the resources used at different life stages do not overlap. The majority of marine invertebrates have complex life-cycles, such as separate benthic and planktonic life stages (Eckman 1996). The life-cycle of many Cnidarians is characterized by the occurrence of structurally and functionally different polyp and medusa life forms. Medusae generations are produced by polyps, but these two life forms can occur simultaneously over the course of a year (Ceh et al. 2015; Morandini et al. 2016). One of the taxonomic features of Cnidarians associated with a complex life-cycle is the great diversity of the reproductive strategies these animals employ, with asexual reproduction playing an important role (Fautin 2002). As asexual reproduction is typically less costly than sexual reproduction, the clonal population can increase rapidly. The presence of a large number of clones, which can initiate sexual reproduction, contributes greatly to the pool of sexually produced propagules. Asexual reproduction enables many Cnidarian species to form dense populations. In extreme cases, the high population density among medusa-like Cnidarians can result in the so-called “jellyfish blooms,” which occur mainly in estuaries and along the coasts of temperate and boreal regions. Jellyfish populations undergo seasonal fluctuations, with sudden outbursts being followed by population crashes. The large aggregations of medusae are typically formed by Scyphozoa and Hydrozoa.

Hydromedusae (Hydrozoa) often dominate mesozooplanktonic medusae communities (Costello and Mathieu 1995). They have a two-stage life-cycle consisting of a small medusa (usually less than 1 cm in size) and a sessile polyp, which is often colonial. In most species, polyps reproduce asexually, whereas medusae reproduce sexually (Bouillon et al. 2004). A small number of hydromedusae are known to produce clonal medusae by asexual budding (Kawamura and Kubota 2008). As budding hydromedusae can be male and female, a relatively high population density is needed to ensure that individuals can find a mate (Kawamura and Kubota 2008). Within the group of budding hydromedusae, there is a sub-group of crawling hydromedusae represented by two genera: *Staurocladia* and *Eleutheria*. They do not swim, but creep on algal substrate, and reproduce asexually by binary fission or by budding (Hauenschild 1956; Hirano et al. 2000; Mills and Hirano 2007).

One of the best-studied species within this group is *E. dichotoma.* As the medusae of this species are hermaphrodites with self-fertilization capabilities (Hauenschild 1956), they do not need partners for mating. Mature medusae tend to favor sexual over asexual reproduction in response to different environmental stressors: e.g., toxicity (cadmium), low temperature, and food scarcity (Schierwater and Hadrys 1998). Young medusae reproduce asexually by budding, but after reaching sexual maturity (at the age of 2–4 weeks), medusae continue to reproduce both sexually and asexually. Increased investment in sexual reproduction is often a response to a deterioration in conditions in a seasonal or a highly variable environment. This strategy leads to the production of more robust or mobile life forms that can overcome or escape unfavorable conditions (Stearns 1976; Kleiven et al. 1992; Schierwater and Hadrys 1998). *Eleutheria dichotoma* seems to be adapted to environmental variation, as it is a cosmopolitan species that has been found in many locations along the Atlantic coasts of Europe, the Mediterranean Sea, and the Black Sea (Schuchert 2009); and that was recently introduced to Australia (Fraser et al. 2006).

The seasonal occurrence of medusae is related to seasonal changes in the biomass of macroalgae, which are their substrate for crawling (Schierwater and Hauenschild 1990). The highest population densities (50 individuals per 10 cm^2^ on average, with a maximum of 107 individuals per 10 cm^2^) were observed on *Ulva* substrate exposed to intensive wave action. Medusae have also been found in sheltered habitats (rock pools), albeit in lower densities (Fraser et al. 2006). *Eleutheria dichotoma* might theoretically predominate in such small water reservoirs with well-defined boundaries, as the relatively high water temperatures in these reservoirs may facilitate asexual reproduction (Ma and Purcell 2005; Kawamura and Kubota 2008). The differences in densities observed among the medusa populations in the Mediterranean Sea and along the east coast of Australia can be explained by the progressive invasion of *E. dichotoma* in Australia. Surprisingly, in their natural habitats in the Mediterranean Sea, medusae have always been observed in very low densities (Schierwater and Hauenschild 1990). This finding suggests that there may be mechanisms that regulate population size.

While the previous laboratory studies have indicated that there are no significant reproductive mode preferences among high-density populations (Schierwater and Hauenschild 1990; Schierwater and Hadrys 1998), the overall reproductive efforts of individual medusae have not previously been examined. *Eleutheria dichotoma* is self-fertilizing hermaphrodite, and its embryos initially develop in a brood pouch. The production of the two types of gametes by hermaphroditic medusae and the production of vital larvae capable of metamorphosis may be a substantial energetic investments made by mature medusae (Kaliszewicz and Lipińska 2013).

The goal of our study was to investigate the strategies of survival and reproduction of medusae maintained in a gradient of population density. In a long-term experiment with controlled food conditions, we compared the survival and reproductive strategies of medusae maintained at three levels of density. We applied a high number of replicates in each experimental regime (roughly 100 individuals per treatment), which allowed us to reliably determine the mortality patterns, including the rate of aging and the hidden heterogeneity in mortality rates. We expected to find differences in the growth and the reproductive rates. Medusae reared in high densities should have a slower growth rate, reduced asexual reproduction, and increased sexual reproduction; characteristics that could be indicative of a strategy for escaping from the overcrowded environment. In our experiment, the intraspecific competition due to increased population density, as well as the large investments in the production of vital larvae, could be expected to result in an increased rate of aging, and thus in a shorter medusae lifespan.

## Materials and methods

### Study organism

The laboratory sample came from the Laboratory of ITZ Ecology and Evolution, Tierärztliche Hochschule Hannover, Germany. Since 2010, the sample has been cultured in the Laboratory of Evolutionary Biodemography at the Max Planck Institute for Demographic Research in Rostock. The original stem culture was collected by Schierwater from Banyuls-sur-Mer on the Mediterranean coast of France (Schierwater and Hauenschild 1990; Ender 1997). The culturing conditions were modified following Hauenschild (1956), Schierwater and Hadrys (1998), and Ringelhan (2015).

### Culturing conditions

The stem culture of the strain $\Omega$ was maintained at a temperature of 23 °C and in a 16D:8L light regime provided by incubators (RUMED 3001 and BINDER). As the source of light, Osram Lumilux Cool Daylight lamps (L18 W/865) were used. The light intensity was around 7–20  $\upmu$mol/m^2^/s (PFD = photosynthetically active photon flux density). These conditions provided a low-intensity light level that resembles the illumination conditions experienced by *E. dichotoma* in its natural environment. The laboratory culture was maintained in salinity 35‰, which reflects the average salinity of marine waters. The culturing medium was prepared from artificial ocean salt (Aquarium Systems Crystal Reefs) and MilliQ water. To reduce the number of foraminifers and the production of biofilm in culturing dishes, the medium was filtered through paper filters (0.5 $\upmu{\text{m}}$ pore diameter, Roth). The colonies of polyps were reared in 70 ml of medium in glass Boveri dishes. The laboratory culture was fed ad libitum two times per week 2-day-old nauplii of brine shrimp (*Artemia salina)*. The medusae from each treatment were maintained on 6-well polycarbonate plates filled with 9.5 ml of medium. The medium in the wells was changed three times per week, always after a feeding. Each medusa in the experimental regimes was fed individually 4–5 nauplii of *Artemia * sp. (food in abundance).

### Experimental design

To create a large sample of primary medusae of the same age within a short period of time, we assembled individuals from four colonies of polyps. The colonies were raised from four larvae produced by a single medusa of a clone Ω. After settling on the substrate, the larvae metamorphosed into the polyp phase. In the first weeks of life, the primary polyps were fed *Artemia* nauplii that had been crushed. When the first stolon branch appeared, polyps were fed living *Artemia * nauplii, ad libitum. The colonies were checked every day for newly released primary medusae. All the medusae produced by the four colonies of polyps were assembled into one collective Boveri dish. Next, single medusa was randomly distributed to wells of polycarbonate plates representing different experimental regimes.

The experiment consisted of three combinations of treatments:

$${\text{D}}. 1:{\text{ 1 Medusa in a well }}\left( { 90 {\text{ wells}} = {\text{ 90 ind}}.} \right),$$

$${\text{D}}. 10:\; 10{\text{ Medusae in a well }}\left( { 11{\text{ wells }} = { 1}10{\text{ ind}}.} \right),$$

$${\text{D}}. 2 5:{\text{ 25 medusae in a well }}\left( { 5 {\text{ wells }} = { 1}25{\text{ ind}}.} \right).$$

The medusae were photographed in experimental wells on their day of birth, and again on the day, they reached sexual maturity (when embryos were noticed inside the umbrella). The photographs of the medusae were used to measure the diameter of the umbrella as a distance between two opposite ocelli, the diameter of the buds, and the diameter of the tentacle knobs. The measured parameters were used to calculate the total size (area) of the vegetative and sexually mature medusae (Schierwater 1989) (see supplementary materials, Figs. A1, A2, A3). All the experimental wells with medusae were checked under a binocular three times per week. The total number of medusae in each well was recorded and the newly released buds were removed. It was sometimes difficult to distinguish between the newly released buds and the individual medusa. We based the distinctions that we made on our earlier observations. Buds are usually smaller and more orange than the medusa mother, and they generally do not have embryos in the umbrella (see the supplementary materials, Fig. A4). However, there were cases in which a medusa and its bud were sharing embryos (see the supplementary materials, Fig. A5), or in which the aging medusa mother was absorbed by a developing medusa bud. Such cases, if recognized, were censored at the time of absorption. Deteriorating medusae have had brown inclusions and a deformed umbrella (see the supplementary materials, Figs. A6, A7, A8).

The medusae were checked for the presence of embryos inside the umbrella. Free-swimming planula larvae, which were released after their initial development as embryos inside the umbrella, were counted and moved to the assembling dish for each separate regime. Each medusa from each experimental regime was directly fed 2-day-old nauplii of *Artemia salina* ad libitum three times per week (Monday, Wednesday, and Friday). The medium was changed 1 h after feeding. The medusae that were fully dissolved after a period of gradual deterioration were considered dead. The dead medusae were not replaced. The experiment was finished when all of the medusae in a well died (dissolved). The buds from each experimental regime were collected in separate dishes and cultured in standard conditions (they were fed three times per week *Artemia * naupli ad libidum). Every second week, proteins from the medusa buds were extracted using standard methods (Pijanowska and Kloc 2004). The samples were stored at − 20 °C, and were later used in an analysis of heat shock proteins.

In addition to the main experiments described above, we also performed a preliminary experiment in which we compared the sizes of the larvae in different densities. Every second week, starting on the day when most of the medusae had reached sexual maturity (around day 50 in each experimental cohort), the random sample of larvae (from 10 to 30) from each regime was photographed under high magnification. The length (longer dimension) and the width (shorter dimension) of the larvae were later measured. Because the pictures were taken irregularly, which could lead to a bias, the results are presented in the supplementary materials (Fig. A9).

### Statistical methods

All the analyses were performed in the R language and environment (R Core Team 2017). The survivorship was analyzed using the Kaplan–Meier estimator (Klein and Moeschberger 2003). The medusae that were lost or absorbed by a bigger bud were treated as censored. The equality of the survival distributions was tested using a log-rank test (Mantel 1967; Cox 1972) (survival R-package). The trend in survival distributions was analyzed using a Gehan–Mantel Trend Test (Leissen et al. 2009).

The empirical mortality data were fitted with *γ*-Gompertz and *γ*-Gompertz-Makeham models (Gompertz 1825; Makeham 1867; Vaupel et al. 1979; Missov et al. 2016; Dańko et al. 2017b) by means of a maximum-likelihood approach. The hazard rate for *γ*-Gompertz-Makeham is defined according to the formula:

1 $$\mu = \frac{{ae^{bx} }}{{1 + \frac{a\gamma }{b}\left( {1 - e^{bx}} \right)}} + c,$$

where *a* is initial age-dependent mortality, *b* is the rate of aging, *γ* is heterogeneity parameter, and *c* is age-independent mortality. For *c* = 0, the model is simplified to a *γ*-Gompertz model, and *a* becomes the intercept of the mortality curve. For each of the populations, we checked the significance of the Makeham term using a likelihood ratio test (LRT). In this case, a pair of models was compared, with one model being nested within the other. Because the null hypothesis *c* = 0 lies at the boundary of the parameter space, we corrected the *p* values by dividing them by two (Ota et al. 2000; Pietrzak et al. 2015). As we found that Makeham term *c* was insignificant in all cases (D1: ratio = 0, *p* = 0.5; D10: ratio = 0.5885, *p* = 0.2215; D25: ratio = 0.3571, *p* = 0.2751), we used *γ*-Gompertz for the rest of the analyses.

Similarly, we used an LRT to compare the *γ*-Gompertz parameters in the three studied populations. An LRT is typically used to compare a pair of models in which one model is nested within the other. Here, we constructed an extended model for three populations (Pletcher 1999). A null hypothesis assumes that one of the corresponding parameters is fixed for all populations, while the alternative hypothesis assumes that each parameter is fitted independently.

The lifetime mean reproductive rates were analyzed using the GLM with Poisson errors. In the model, we included the total number of buds as a count-dependent variable, and the log of exposures was calculated as a sum of the lifespans of individuals (as an offset). The normality of the residuals was checked using quantile–quantile plots.

The age-specific reproductive rates for both sexual and asexual reproduction were smoothed by fitting one-dimensional Poisson penalized splines (P-splines). The procedure was performed using the *MortalitySmooth* R-package (Camarda 2012). The optimal values of the smoothing parameter were selected using the BIC model selection, and the fitting allowed for the potential over-dispersion. Standard errors for the reproduction rates were calculated using the delta method from the obtained standard errors of the smoothed log-reproduction rates. The piecewise confidence intervals for the fitted reproductive rates were calculated from the abovementioned standard errors with a normality assumption.

The diameter of the umbrella, the diameter of the buds, and the diameter of the tentacle knobs were used to calculate the two-dimensional surface area of the asexual and sexual medusae, which is a better indicator of size for very small organisms (Schierwater 1989). To estimate the size of a 1-day-old vegetative medusae, we analyzed the area of their body, calculated as follows:

2 $$\begin{aligned} {\text{Area}}_{{{\text{veg}}}} & = 2.622\,{\text{Umbrella}}\,{\text{diameter}}^{{2.26}} \\ & \quad + \,\sum {\left( {0.265\;{\text{bud}}\,{\text{diameter}}^{{2.239}} } \right)\left( {{{\upmu}}{\text{m}}^{2} } \right)} . \\ \end{aligned}$$

The formula was updated for sexually mature medusae by including the area of six tentacle knobs:

3 $$\begin{aligned} {\text{Area}}_{{{\text{sex}}}} & = 2.622\,{\text{Umbrella}}\,{\text{diameter}}^{{2.26}} \\ & \quad + \,6 \,{\left( {714.9\;{\text{tentacle}}\,{\text{knob}}\,{\text{diameter}}^{{0.955}} } \right)\left( {{{\upmu}}{\text{m}}^{2} } \right)} . \\ \end{aligned}$$

In the analysis of the sizes of the larvae, the length and the width were treated as multivariate-dependent variables, and the density was treated as a continuous covariate. The analysis was performed with MANCOVA (*car* R-package, Pillai method), assuming a type III sum of squares (significant interaction, Fig. A9).

## Results

### Patterns of survivorship and mortality

**Fig. 1:**
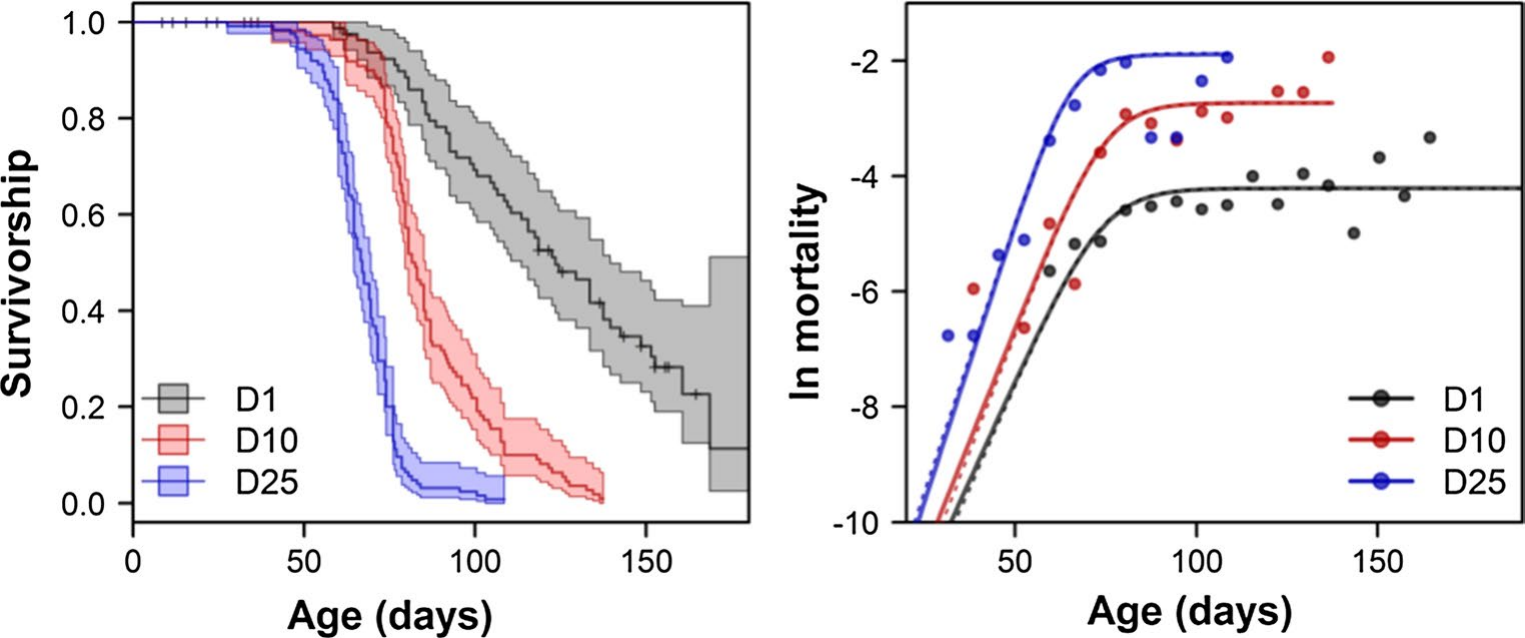
Survivorship of medusae and logarithmized mortality rates at different densities. The survivorships (left panel) were based on the Kaplan–Meier estimator of survival. Marker “+” represents censoring events (medusae lost or absorbed by a bigger bud). The empirical mortality rates (right panel) were fitted with a *γ*-Gompertz model of mortality using a maximum-likelihood method. The solid lines represent the fit of the most parsimonious model that assumes a common *a* parameter for all densities, whereas the dashed lines (which overlap considerably with the solid lines) represent each population that was fitted separately by this model. The empirical mortality rates are represented as dots that were calculated from 7-day aggregated data

We found significant differences in the survivorships of the three density regimes (log-rank, *χ*^2^ = 253.4, *p* < 0.0001, Fig. 1, left panel). The lowest survival rates were in the highest density conditions (25 individuals in a well, D25), while the highest survival rates were in the lowest density conditions (one individual per well, D1). These results were confirmed by the trend test (Gehan–Mantel test, *Z* = − 14.66, *p* < 0.0001). The empirical mortality rates were fitted well by a *γ*-Gompertz model (Vaupel et al. 1979; Missov et al. 2016), and were reflected by curves indicating an exponential rate of growth early in life, which then decelerated and reached a plateau at older ages (Fig. 1, right panel). The three curves were similar in the intercept parameter *a* (LRT, ratio = 0.1224, *p* = 0.9406, *a* = 5.26 × 10^−7^), but differed in the rate-of-aging parameter *b* (LRT, ratio = 8.270, *p* = 0.0160) and the heterogeneity parameter *γ* (LRT, ratio = 11.04, *p* = 0.0040). The population-specific heterogeneity decreased with population density (D1: *γ* = 9.31, D10: *γ* = 2.41, and D25: *γ* = 1.28), whereas the rate of aging increased with population density (D1: *b* = 0.139, D10: *b* = 0.157, and D25: *b* = 0.193). Qualitatively and quantitatively similar results were obtained when each population was fitted separately (Fig. 1, right panel, dotted lines). A comparison of the expression of heat shock proteins by medusa buds revealed no significant differences between different regimes of density (*F* = 0.0087, *p* = 0.9913).

### Reproduction rate

**Fig. 2:**
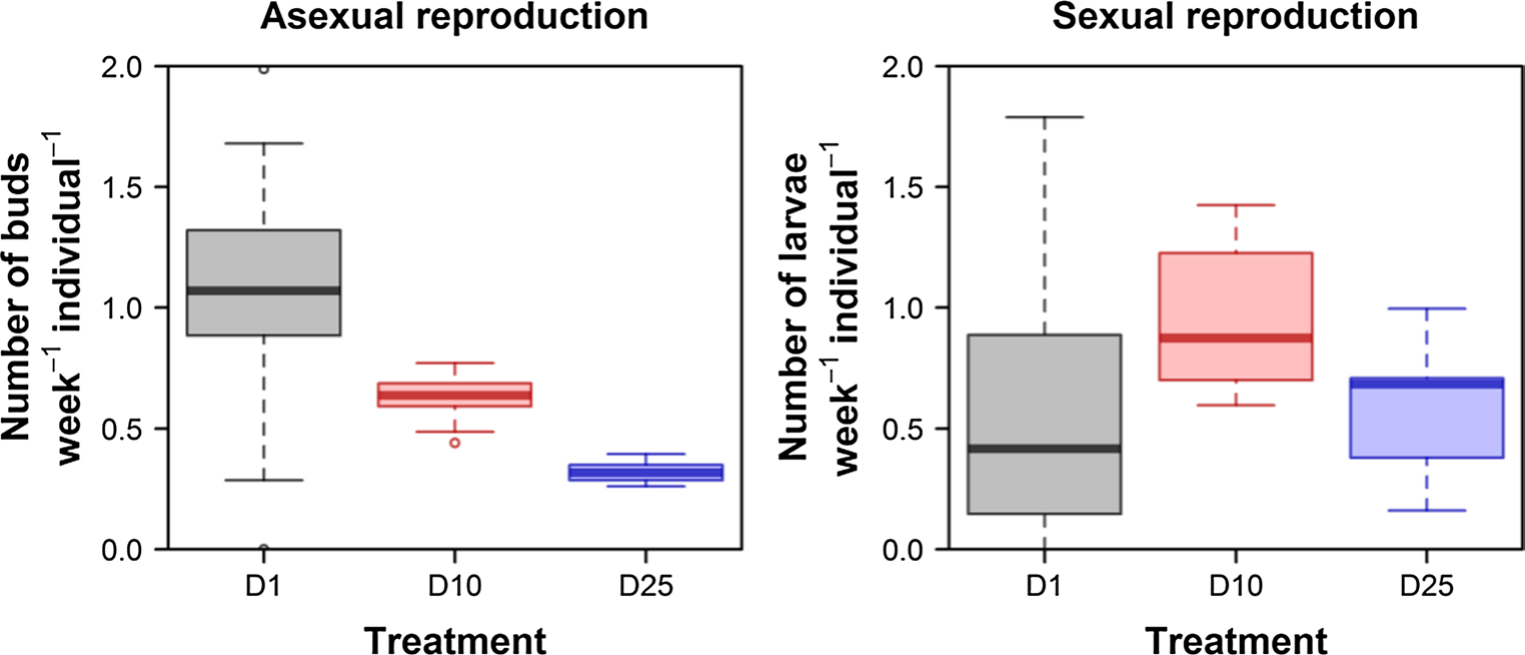
Asexual and sexual reproduction of medusae maintained in different densities. The box plots represent reproduction rates counted as a sum of released buds divided by the sum of the lifespans of all of the individuals. All the medusae were fed according to the same feeding regime (*Artemia* ad libitum)

**Fig. 3:**
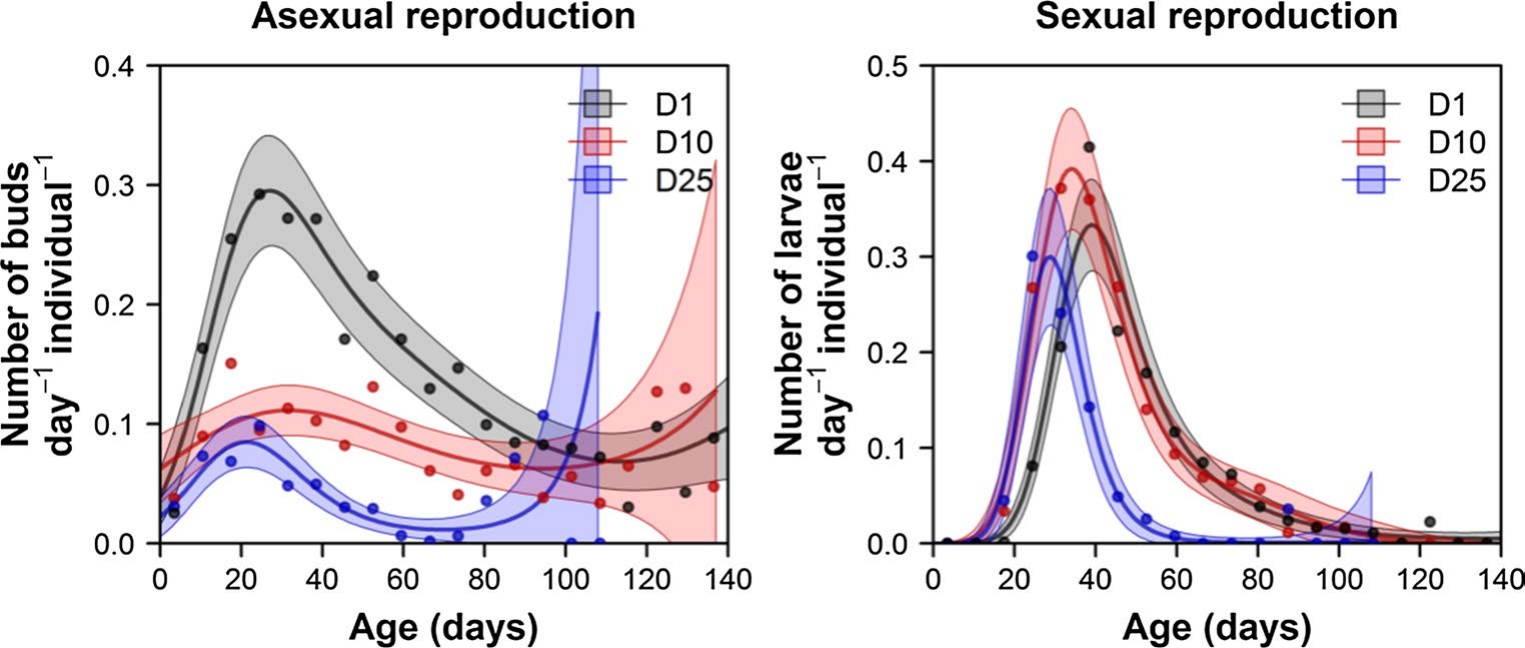
Age-specific asexual (**a**) and sexual (**b**) reproduction rates. The colors represent different regimes of density: gray (one medusa in a well), red (10 medusae in a well), and blue (25 medusae in a well). The increased asexual reproductive rate of D25 in the higher age classes might be the effect of decreased density in experimental wells due to deaths (dead individuals were not replaced) (but see Discussion)

The asexual reproduction rate decreased with density (GLM, Poisson errors, density as continuous predictor, *χ*^2^ = 151.2, *p* < 0.0001). The medusae that were maintained individually had the highest asexual reproduction rate, whereas the medusae that were reared in the highest density conditions had a rate that was 3.5 times lower (Fig. 2, left panel). Furthermore, the variation in the reproduction rate between individual medusae was much lower in D10 and in D25 than in D1 (coefficient of variation, D1: 0.341, D10: 0.160, and D25: 0.164). Density also influenced the sexual reproduction rate (GLM, Poisson errors, density as categorical predictor, *χ*^2^ = 1047, *p* < 0.0001), but there was no linear trend (Fig. 2, right panel). We applied multiple comparisons of means (Tukey’s contrasts) as a post hoc to the fitted model. The highest rate of larvae production was observed in the medusae maintained in D10 (0.962 larvae per medusa per week). This rate was 1.577 times higher than in D1 (*z* = 3.170, *p* = 0.0042), and it was 1.637 times higher than in D25 (*z* = 3.248, *p* = 0.0033). There was no significant difference in the larvae production rate between D1 and D25 (*z* = 0.0226, *p* = 0.9722). The age-specific asexual reproduction rate changed with age in all three regimes of density (Fig. 3). The reproduction rate of the medusae that were maintained individually displayed a clear pattern: the rate increased at the beginning of life, peaked roughly between the 20th and the 30th day of life, and decreased later in life. A similar pattern, but with a lower peak reproduction rate, was observed in regime D25. The initial peak in the reproduction rate was lowest in regime D10. For the medusae from all of the regimes, asexual reproduction continued over the whole life course, and even increased late in life. In all of the density regimes, the pattern of the age-specific sexual reproduction rate was similar to that of the asexual reproduction rate. The rate of reproduction increased at the beginning of life, reached a peak between the 30th and the 40th day, and then decreased with age. Sexual reproduction ended early and well before the end of the medusa’s life. The medusae from regimes D10 and D25 tended to initiate sexual reproduction earlier than the medusae from D1.

### Sizes of the medusae at birth and at sexual maturation

**Fig. 4:**
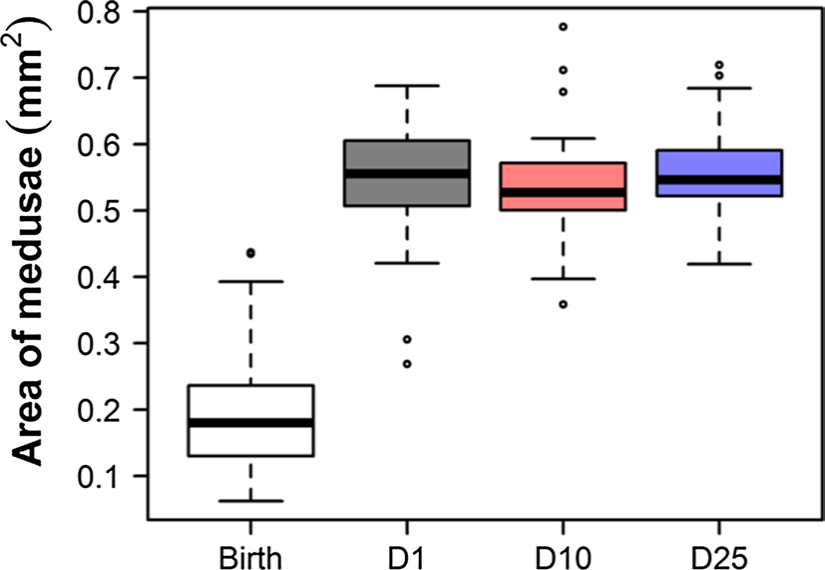
Area of the medusae at birth and at sexual maturation, reared in different densities. The area of the medusae was calculated according to Eq. 1 (Birth) and Eq. 3 (D1, D10, and D25)

The analysis of variance (ANOVA) showed significant differences between the average area of medusa (Eq. 2) at birth and the average area (Eq. 3) at sexual maturity for each density (*F* = 453.5, *p* < 0.0001). The average area of the sexually mature medusae in the different densities was roughly 2.8 times bigger than the average area recorded at birth (Fig. 4, Tukey’s HSD, the area at birth compared with every density always gives *p* < 0.0001). However, the average areas of the sexually mature medusae did not differ between the regimes (Tukey’s HSD, D1–D10: *p* = 0.7470, D1–D25: *p* = 0.9942, D10–D25: *p* = 0.6071).

## Discussion

### Reproduction

Our results show that there is an optimal population density in laboratory conditions, which is supported by the fact that the sexual reproduction rate was the highest in the moderate-density conditions (D10). The increase of investments in sexual reproduction seemed to occur at the cost of asexual reproduction as there was a significant decrease in the budding rate with density. However, further investigations are needed to test if such a trade-off really exists, especially that the negative effects of overcrowding at the highest population density are likely to overlay the positive effects on sexual reproduction. It must be noted that it is unlikely that the increased rate of sexual reproduction at D10 is a consequence of cross-fertilization with other individuals (note that in D1, only single individuals were present). In *E. dichotoma*, eggs and sperm are produced simultaneously by the same medusa, and fertilization takes place within the brood pouch, where the embryos develop into planula larvae. The larvae are released by a tearing of the umbrella, which subsequently regenerates before releasing another set of larvae (Hauenschild 1956).

Our results show that the age-specific rates of asexual reproduction have unexpected patterns in late-life stages (Fig. 3). While the sexual reproduction rate in all of the density regimes followed a typical senescence pattern and decreased with age, the asexual reproduction rate tended to increase later on (Fig. 3, left panel). This pattern can be explained by an extraordinary feature of *E. dichotoma* medusae, whereby a developing bud may overgrow the aging medusa mother, and can sometimes even absorb it. This phenomenon may be responsible not only for the observations of increased medusae late-life reproduction rate, but also for the prolongation of their lifespans. Even if we had censored all of the medusae in our experiment at the point in time when they exhibited absorption (observed only in D1), it is possible that some of absorptions would have gone unnoticed. Finally, the increased asexual reproductive rate at later ages might have also been an effect of decreased density in the experimental wells due to deaths (as the dead medusae were not replaced) or the effect of sampling noise (as few individuals were left; notice the widening confidence intervals in Fig. 3).

At all density regimes, there is a peak of the sexual and vegetative reproduction rate at early life stages. The medusae that were maintained individually reached their highest age-specific asexual reproduction rate at the beginning of life, and their highest larvae production rate shortly thereafter. Our results suggest that under higher density conditions (D10 and D25), medusae may reach their peak sexual reproduction rate slightly earlier, and could reach sexual maturity earlier, as well (Fig. 3, right panel). However, both population densities have no detectable effects on size at maturity (Fig. 4). All the medusae used in this experiment were directly detached from polyps. These so-called primary medusae emerge early in the spring, when asexual reproduction of polyps is triggered by increasing temperatures. The very early peaks in the life course of both sexual and asexual reproduction may be attributable to a process of adaptation to the large amount of food available at the beginning of the vegetation season. The field studies from temperate regions have shown that the highest levels of diversity and density of different species of hydromedusae are reached in the late winter/early spring period, which coincides with the yearly maxima of phytoplankton and zooplankton (Costello and Mathieu 1995).

Medusae maintained at medium and high population density reached sexual maturity earlier at the costs of smaller size of their offspring (planula larvae). Density did not affect size at sexual maturity (Fig. 4), even though medusae D25 and D10 seemed to mature slightly earlier than medusae D1 (Fig. 3, right panel). This result suggests that the medusae that experienced medium and high densities may have had higher numbers of embryos or bigger embryos in brood pouches. While the latter has not been confirmed (larvae produced in D10 and D25 were actually smaller at the beginning of experiment, Fig. A9), our finding that the production of larvae was higher in moderate-density than in low-density conditions at least partially confirms the first hypothesis (Fig. 2, right panel). We observed that in high/moderate densities, both the medusae buds (Fig. A4, Dańko, pers obs) and the larvae tended to be smaller (Fig.A9). However, the sizes of the larvae increased with the age of the medusae (Fig. A9). This increase is likely a byproduct of the experimental design, as the population densities in the research vessels (and their effects on life histories) decreased with time. The production of bigger larvae may be adaptive, because bigger larvae could have more energy reserves that allow them to spend more time swimming before settling. Thus, bigger larvae may be more likely than smaller larvae to find a habitat suitable for a wider dispersion (Marshall and Bolton 2007). The production of smaller larvae in D10 and D25 might be associated with earlier sexual maturity among the medusae (Fig. 3, right panel). It is likely that the medusae maintained a high individual growth rate at the expense of offspring size. Clearly, further study is needed to investigate the effects of population density and other environmental factors on the sizes of the larvae and the medusa buds.

### Survival

Our study showed that high population density levels had clear effects on the survival patterns of medusae in laboratory conditions. As the density level increased, the medusae lived shorter lives, and had an increased rate of aging. There are two possible explanations for these patterns, which are not mutually exclusive. The faster rate of aging could be (i) an outcome of direct negative effects of density on life-history traits, such as via the accumulation of toxic metabolites in wells; or it (ii) could be an indirect effect of trade-offs between investments in sexual reproduction and in tissue maintenance. Both explanations imply that there is a link between population density and individual deterioration, and hence senescence. Senescence is generally defined as a decrease in the reproduction rate and an increase in the mortality rate with age (Dańko et al. 2015; Schaible et al. 2015). In our experiment, at every density level, the rise in mortality was accompanied by physiological deterioration. However, the patterns of deterioration depended on the degree of density. After reaching its highest sexual reproduction rate, the medusae maintained in high population densities (D25) had an empty and expanded umbrella with short tentacles (see Picture A6). This outcome could reflect the direct costs of the increased rate of sexual reproduction, including overcrowding; which could in turn be linked to an increased rate of aging. Interestingly, medusae reared under medium-density conditions (D10) did not show a pattern of deterioration, which suggests that this level of density is more tolerable for the species. Like the medusae maintained individually (D1), medusae D10 shrank with age, and eventually dissolved.

The mortality rates decelerated at later ages, which may have been an outcome of (i) decreasing population density with time, or/and (ii) hidden heterogeneity in levels of individual frailty (Carey et al. 1995; Dańko et al. 2017b; Missov et al. 2016; Vaupel et al. 1979). The individuals of a single population are likely to differ in their susceptibility to all causes of death, even if the population is genetically homogenous. On one hand, even tiny differences between individuals can lead to a substantial degree of heterogeneity in stressful conditions, but on the other, stressful conditions can cause less robust individuals to be selected out of the population more quickly. The fitted model showed that the highest degree of heterogeneity in unobserved mortality risks was present in the lowest density conditions. This finding can be explained by the high degree of individual variation in resource allocation strategies, and is supported by the higher degree of variation in other traits (i.e., asexual and sexual reproduction rates, Fig. 2).

The decrease in the density level with each experimental treatment did not lead to a convergence of mortality rates across the different experiments. We observed that the mortality plateau was highest in the highest density conditions, and was lowest in the lowest density conditions. There are two potential explanations for this effect: (i) that differences in population’s hidden heterogeneity greatly shape mortality patterns later in life; and (ii) that the harmful effects experienced early in life in high-density conditions cannot be overcome later in life, when density substantially decreases. This second explanation is clearly supported by the increase in the rate-of-aging parameter with population density (see patterns of survivorship and mortality, results section).

Finally, our results also revise recent findings. Ringelhan (2015) suggested that the mortality pattern of medusae follows a “hump shape” characterized by decreasing mortality in later age classes. This shape of mortality is probably both an artifact of the much smaller sample sizes used, and a result of the inclusion in the mortality analysis of medusae that had absorbed buds. Differentiating between the medusa mother and the medusa bud is sometimes challenging, and can be especially difficult in experiments that include multiple generations (Dańko et al. unpubl data). In this experiment, we studied density effects in the first generation of medusae (primary medusae) only, which presumably limited the number of controversial cases.

## Conclusions

The ability to reproduce asexually enables budding medusae (Hydrozoa: Capitata) to occur in high population densities in coastal regions (Hirano et al. 2000; Kawamura and Kubota 2008). Sexually reproducing individuals are generally found in favorable environmental conditions, which suggest that the shift from asexual to sexual reproduction is associated with increased energetic demands (Kawamura and Kubota 2008). *Eleutheria dichotoma* is the only known species of budding hydromedusae that simultaneously reproduces both sexually and asexually. However, the modes of reproduction can be modified by environmental conditions (Schierwater and Hadrys 1998). The production of vital larvae that are able to metamorphose into the polyp stage and to settle as a more stable colony may be a substantial energetic investment for sexually mature medusae. As the larvae are lecithotrophic (incapable of feeding themselves) (Collins 2002; Marshall and Bolton 2007), brooding embryos in a brood pouch is a critical means of supplementing resources. The byproduct of this strategy is that larvae get additional chances for dispersal, because medusae carrying embryos may float passively to different environments.

Our results show that medusae may have flexible life-history responses to different population densities. Thus, the reproductive/propagation strategies of medusae may occupy different places along the r/K-strategies’ continuum. In laboratory conditions characterized by low population densities, the animal invests in a strategy of clonal propagation (the medusa stage most closely fits the definition of the r-strategy). However, it attempts to escape unfavorable conditions (e.g., high population densities) by producing offspring (larvae) that are able to disperse and settle a new colony of polyps (the polyp stage most closely fits the definition of the K-strategy). The production of a large number of short-lived planula larvae increases the survival chances of a genet in the polyp stage. In unfavorable conditions, polyps are presumably the main source of medusae in the population, because they are long-lived and more resistant to environmental stress (Dańko et al., unpubl data). Our results show that seasonally occurring medusae are susceptible to variable environmental conditions, which may shorten their lifespan. The higher investments in sexual reproduction come at a cost of lower asexual budding rates and of lower investments in tissue maintenance, which in turn result in increased mortality and aging rates.

Despite some obvious limitations of our study associated with unlimited food availability (the most limiting factor in dense populations) and the gradual decrease in the population size with time (due to the removal of medusa buds and larvae during the experiment), the obtained results contribute to our understanding of the role of population density in shaping life-history traits in natural populations of *E. dichotoma*. Further studies are needed on the effects of population density and other environmental factors on the survival and reproduction strategies of both medusae and colonial polyps.

## Electronic supplementary material

Below is the link to the electronic supplementary material.

Supplementary material 1 (CSV 1 kb)

Supplementary material 2 (CSV 0 kb)

Supplementary material 3 (CSV 1 kb)

Supplementary material 4 (CSV 0 kb)

Supplementary material 5 (CSV 1 kb)

Supplementary material 6 (PDF 965 kb)
