# Supplementary material for: Population density shapes patterns of survival and reproduction in Eleutheria dichotoma (Hydrozoa: Anthoathecata)
Source: Mar Biol. 2018 Feb 17;165(3):48. doi: 10.1007/s00227-018-3309-z (PMC5816771; doi:10.1007/s00227-018-3309-z)
Supplement: Supplementary file 6 — Supplementary material 6 (PDF 965 kb) [file 227_2018_3309_MOESM6_ESM.pdf]

Density shapes patterns of survival and reproduction in hydromedusa *Eleutheria dichotoma*

Aleksandra Dańko[a,*], Ralf Schaible[a], Joanna Pijanowska[b], Maciej J. Dańko[a,*].

[a]Laboratory of Evolutionary Biodemography, Max Planck Institute for Demographic Research, Konrad-Zuse-Strasse 1, 18057 Rostock, Germany.

[b] Department of Hydrobiology, University of Warsaw, Żwirki i Wigury 101, 02-089 Warszawa.

*Corresponding authors: e-mails: adanko@demogr.mpg.de; danko@demogr.mpg.de

Supplementary materials

I. Additional figures

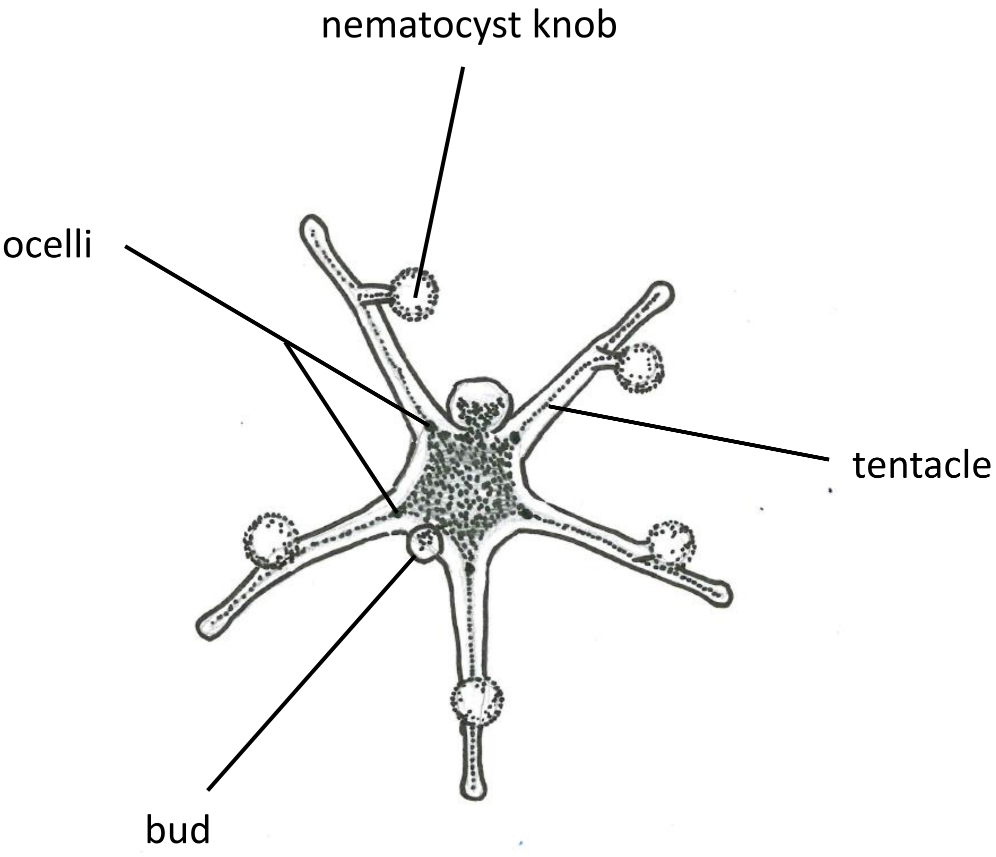

Figure A1. One-day-old medusa of *Eleutheria dichotoma* with developing buds (vegetative medusa) (Dańko, A. 2016)

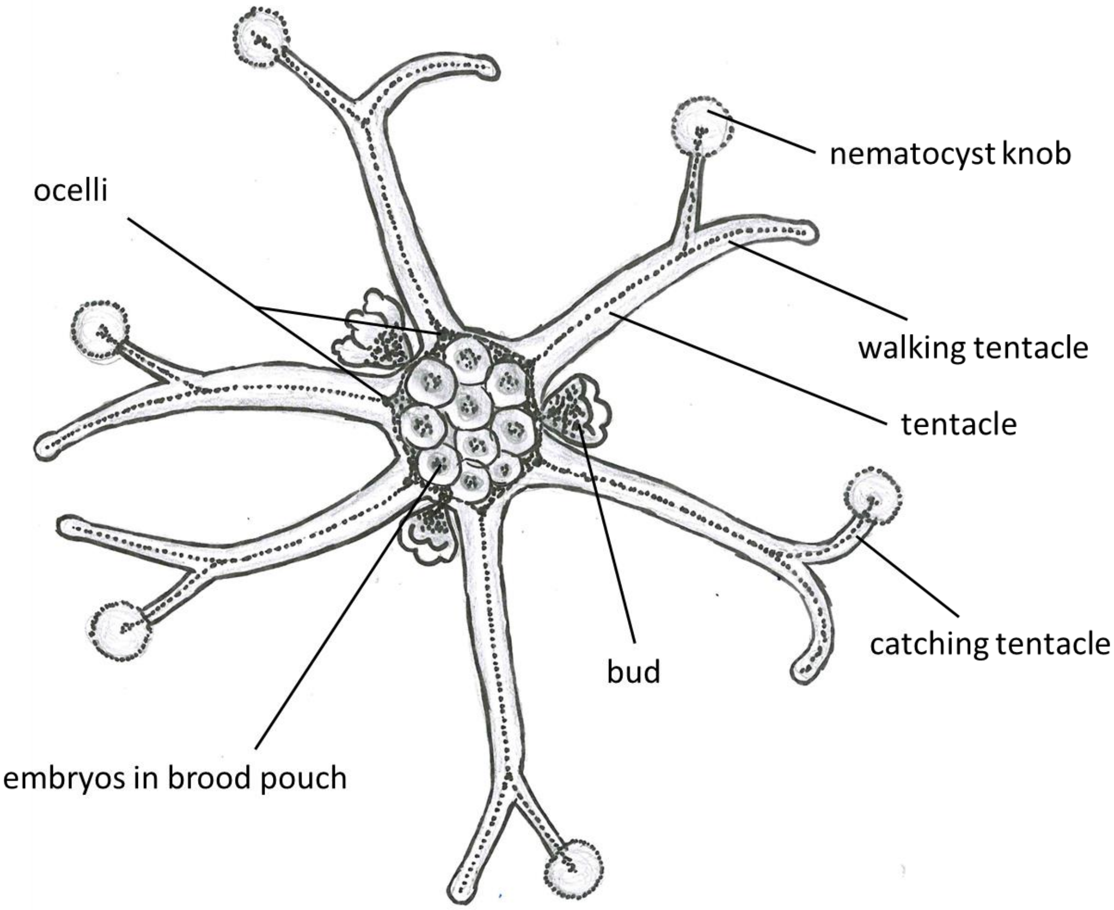

Figure A2. Mature medusa of *Eleutheria dichotoma* viewed from the aboral side, with larvae developing in a brood pouch. About 25-fold magnification (Dańko, A. 2016)

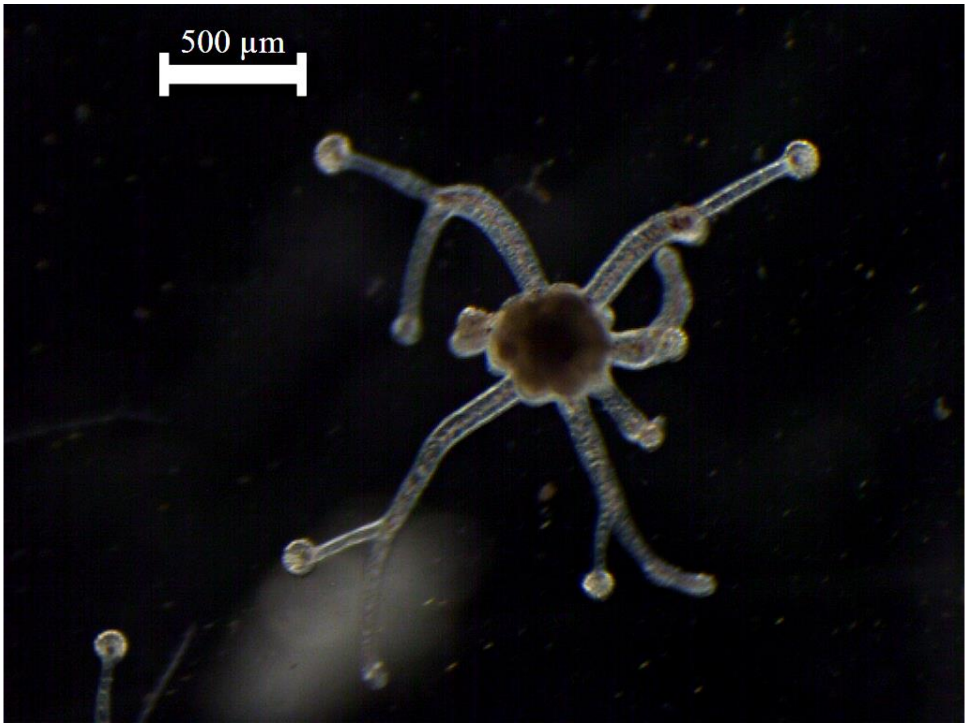

Figure A3. Mature medusa with embryos in the umbrella (from density D25)

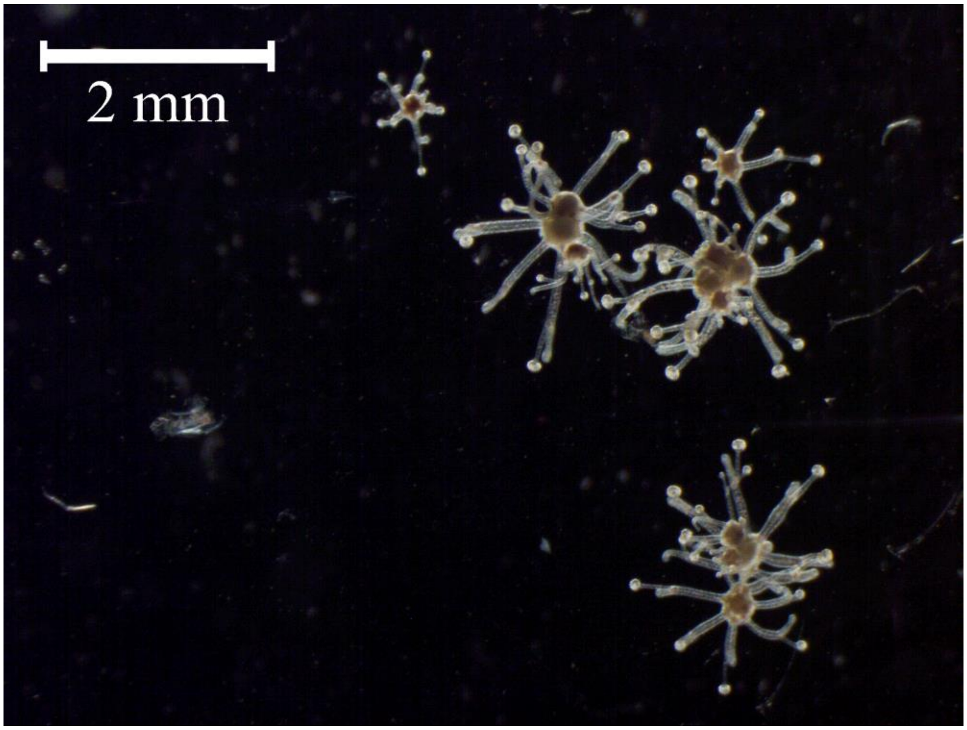

Figure A4. Three experimental medusae and two released buds (the buds are smaller than the medusa mothers)

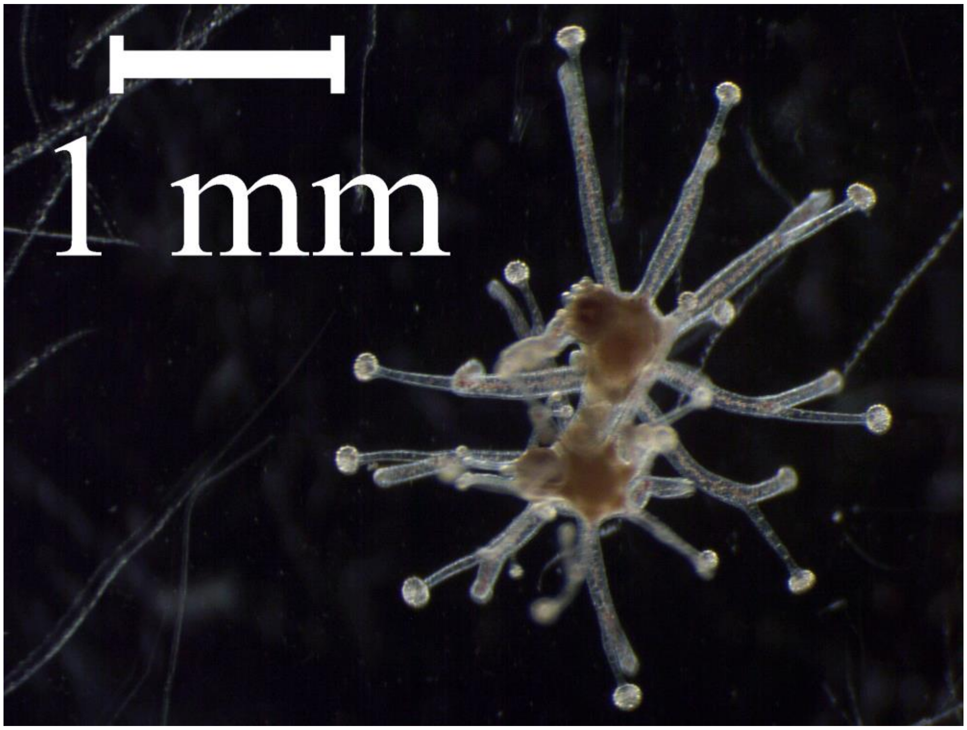

Figure A5. A medusa mother and a bud sharing larvae

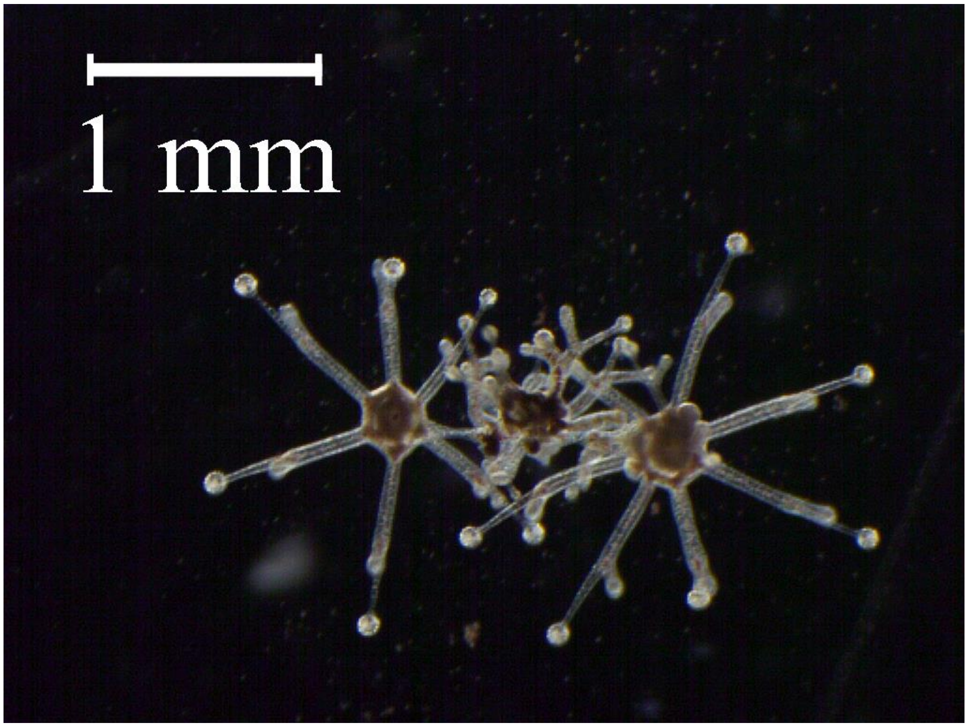

Figure A6. Two young buds and an old mother medusa (in the middle), just before separation

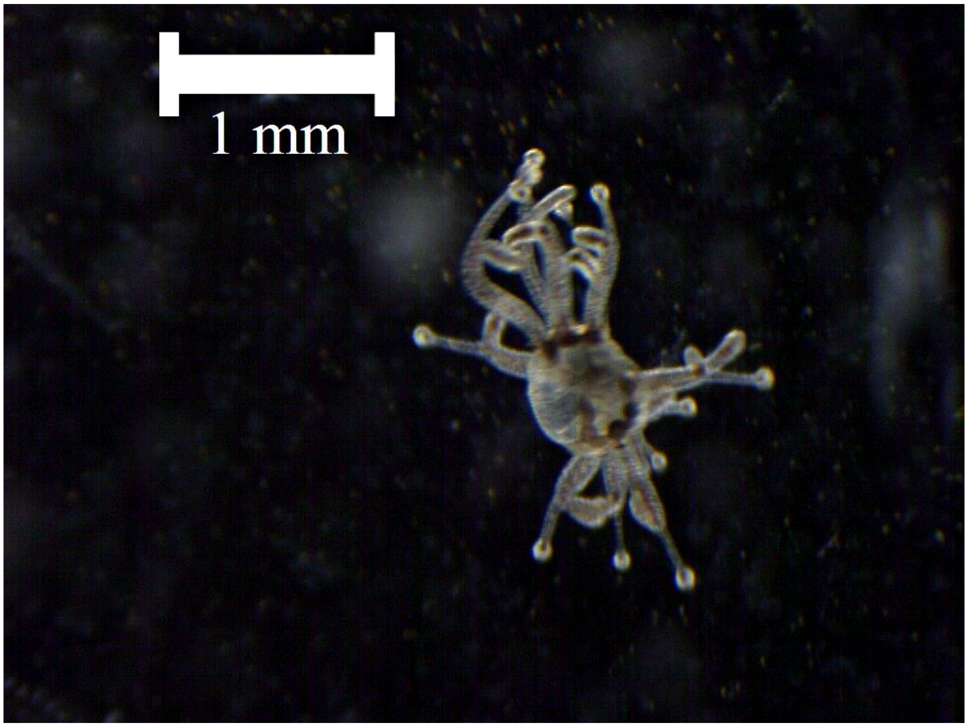

Figure A7. The expanded umbrella of a medusa maintained in density D.25, shortly after the release of larvae

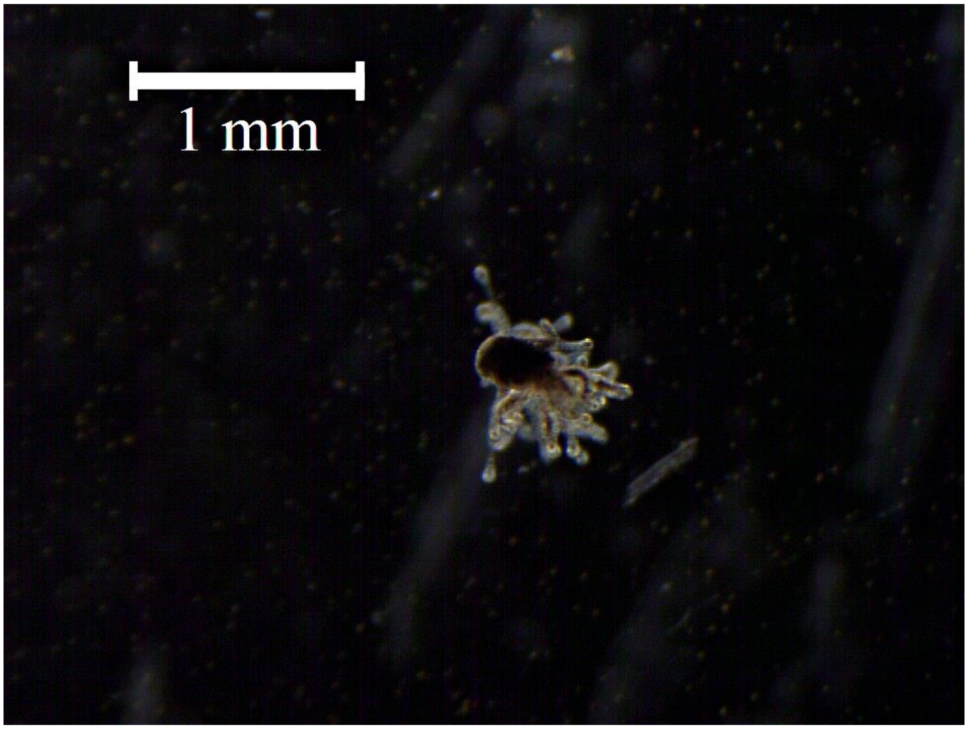

Figure A8. A shrinking old medusa with deformed tentacles

II. Size of larvae

A multivariate analysis of covariance (MANCOVA, type III of sum of squares) showed that there was a significant effect of density on the length and the width of larvae (F= 8.56, p=0.0002). There was, however, no significant main effect of the day of the experiment (F=0.08, p=0.9267), and there was significant interaction between the density and the length/width of the larvae (F=3.80, p=0.0233). The larvae released in densities 10 and 25 were smaller at day 50 of the experiment (when we started measuring their sizes), but their sizes increased with time, eventually reaching the sizes of the D1 larvae. The sizes of the larvae released by the medusae that were maintained individually were roughly constant during the experiment (Fig. A8).

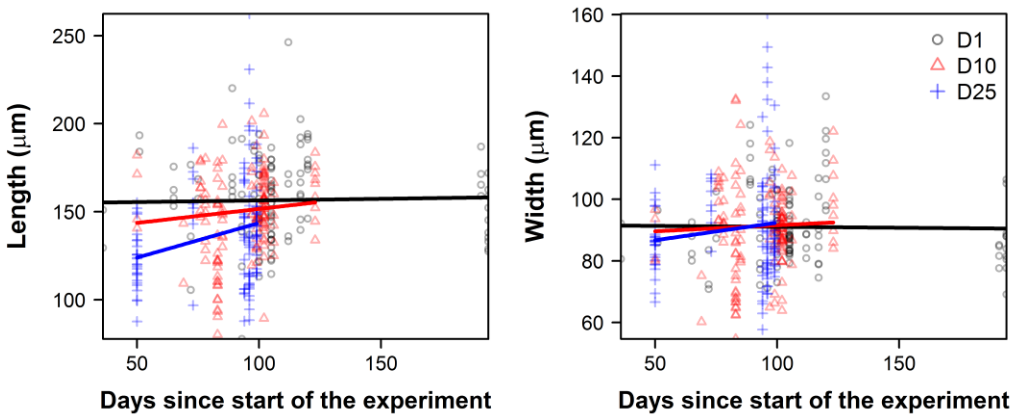

Figure A9. The length (left panel) and the width (right panel) of the larvae released in three density regimes during the experiment. The different markers represent data points for the three different densities; the lines show fitted a model. The length of the lines reflects the range of the observed days since the beginning of the experiment for each density.
